# Supplementary material for: The causal relationship between the human gut microbiota and pyogenic arthritis: a Mendelian randomization study
Source: Front Cell Infect Microbiol. 2024 Nov 26;14:1452480. doi: 10.3389/fcimb.2024.1452480 (PMC11629706; doi:10.3389/fcimb.2024.1452480)
Supplement: Supplementary file 1 [file DataSheet1.zip › mendelian test/ebi-a-GCST90027836.csv_leave_one_out.pdf]

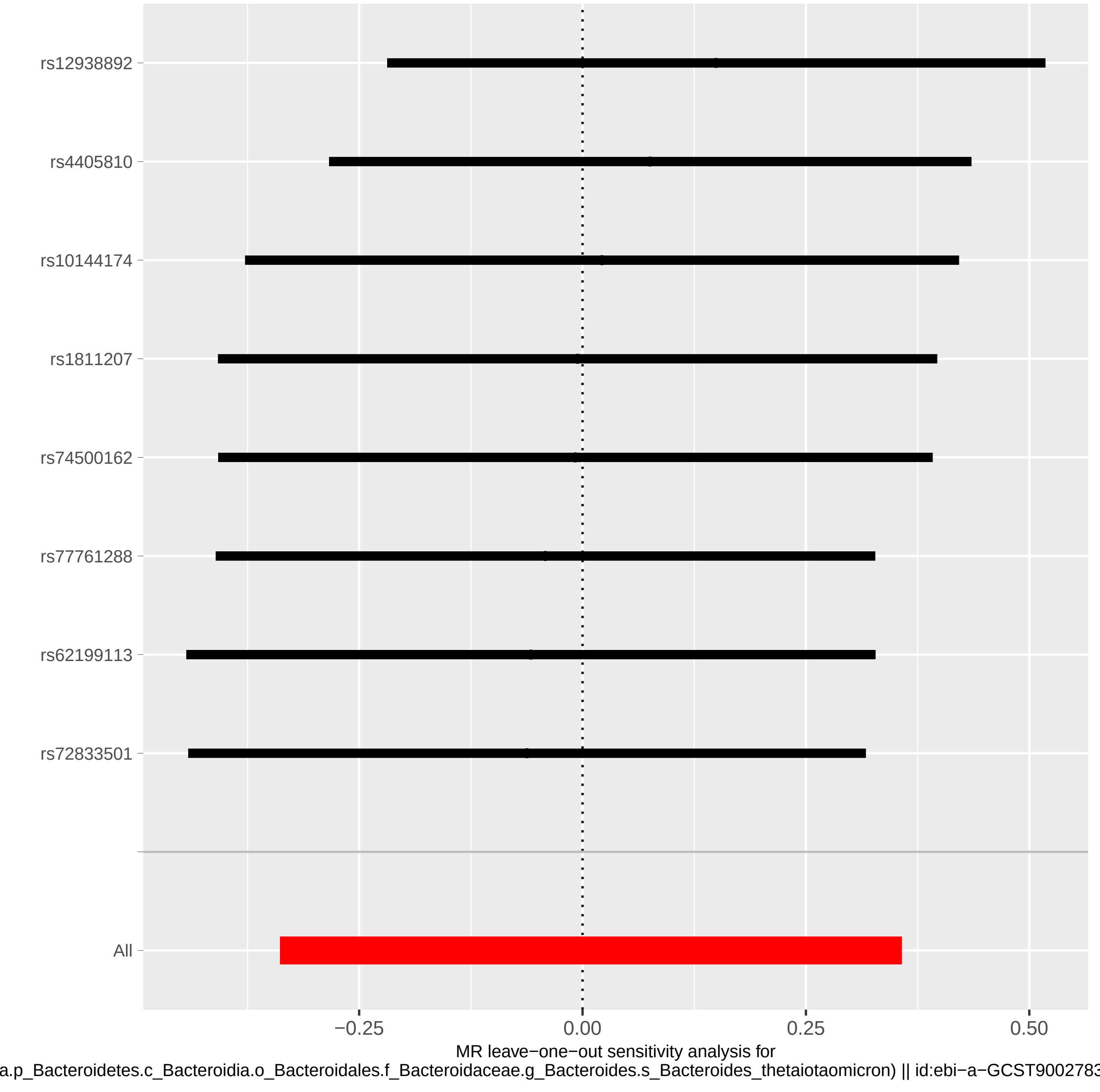

rs12938892
rs4405810
rs10144174
rs1811207
rs74500162
rs77761288
rs62199113
rs72833501
All
−0.25
0.00
0.25
0.50
MR leave−one−out sensitivity analysis for
a.p_Bacteroidetes.c_Bacteroidia.o_Bacteroidales.f_Bacteroidaceae.g_Bacteroides.s_Bacteroides_thetaiotaomicron) || id:ebi−a−GCST900278
